# Supplementary material for: β3-Adrenoceptor redistribution impairs NO/cGMP/PDE2 signalling in failing cardiomyocytes
Source: eLife. 2020 Mar 31;9:e52221. doi: 10.7554/eLife.52221 (PMC7138611; doi:10.7554/eLife.52221)
Supplement: Transparent reporting form [file elife-52221-transrepform.docx]

***eLife’s* transparent reporting form**

We encourage authors to provide detailed information *within their submission* to facilitate the interpretation and replication of experiments. Authors can upload supporting documentation to indicate the use of appropriate reporting guidelines for health-related research (see [EQUATOR Network](http://www.equator-network.org/%20)), life science research (see the [BioSharing Information Resource](https://biosharing.org/)), or the [ARRIVE guidelines](http://www.plosbiology.org/article/info:doi/10.1371/journal.pbio.1000412) for reporting work involving animal research. Where applicable, authors should refer to any relevant reporting standards documents in this form.

If you have any questions, please consult our Journal Policies and/or contact us: [editorial@elifesciences.org](mailto:editorial@elifesciences.org).

**Sample-size estimation**

- You should state whether an appropriate sample size was computed when the study was being designed
- You should state the statistical method of sample size computation and any required assumptions
- If no explicit power analysis was used, you should describe how you decided what sample (replicate) size (number) to use

Please outline where this information can be found within the submission (e.g., sections or figure legends), or explain why this information doesn’t apply to your submission:

Since the response values and deviation values of cells were hard to predict beforehand sample size and replicates were based on prior work using the FRET sensor method and for cell staining.

Retrospective power analysis using mean group values and deviation values of 1 (=a value that is a bit higher than the deviation recorded in most of the experiments performed for this manuscript) shows, that the sample sizes, which were used, with a minimum of ten cells per treatment condition are generally leaving a power of 80 and an alpha value of 0.05, meaning a chance of 5% to detect a false positive.

**Replicates**

- You should report how often each experiment was performed
- You should include a definition of biological versus technical replication
- The data obtained should be provided and sufficient information should be provided to indicate the number of independent biological and/or technical replicates
- If you encountered any outliers, you should describe how these were handled
- Criteria for exclusion/inclusion of data should be clearly stated
- High-throughput sequence data should be uploaded before submission, with a private link for reviewers provided (these are available from both GEO and ArrayExpress)

Please outline where this information can be found within the submission (e.g., sections or figure legends), or explain why this information doesn’t apply to your submission:

Replicates were all of a biological nature, due to the methods used (=FRET and immunocytochemical stainings). The number of animals and single cells are all depicted in the respective figures representing the data. There was no exclusion of data, hence no exclusion criteria.

**Statistical reporting**

- Statistical analysis methods should be described and justified
- Raw data should be presented in figures whenever informative to do so (typically when N per group is less than 10)
- For each experiment, you should identify the statistical tests used, exact values of N, definitions of center, methods of multiple test correction, and dispersion and precision measures (e.g., mean, median, SD, SEM, confidence intervals; and, for the major substantive results, a measure of effect size (e.g., Pearson's r, Cohen's d)
- Report exact p-values wherever possible alongside the summary statistics and 95% confidence intervals. These should be reported for all key questions and not only when the p-value is less than 0.05.

Please outline where this information can be found within the submission (e.g., sections or figure legends), or explain why this information doesn’t apply to your submission:

The statistical analysis is described and justified in the methods section as follows:

**Statistics.** Statistical differences were analysed using OriginPro 8.6 and GraphPad Prism 5 software. Normal data distribution was determined by Kolmogorov-Smirnov test. For the comparison of two independent groups with skewed distribution Mann-Whitney U test was used. For the comparison of normally distributed data, a two-tailed T-test (for comparing morphometric and echocardiographic data from independent animals, Suppl. Fig. 1) or a mixed ANOVA followed by χ2-test (when data from several cells from multiple individual animals were compared) were applied. Differences were considered significant at p-values below 0.05. All data are presented as means ± s.e.m.

Exact p-values are shown in the results text.

Furthermore specific tests used for data sets are in the figure legends right under the corresponding figures for the respective data.

(For large datasets, or papers with a very large number of statistical tests, you may upload a single table file with tests, Ns, etc., with reference to sections in the manuscript.)

**Group allocation**

- Indicate how samples were allocated into experimental groups (in the case of clinical studies, please specify allocation to treatment method); if randomization was used, please also state if restricted randomization was applied
- Indicate if masking was used during group allocation, data collection and/or data analysis

Please outline where this information can be found within the submission (e.g., sections or figure legends), or explain why this information doesn’t apply to your submission:

Cells were treated pharmacologically and allocated into groups according to which treatment their treatment.

n/a for this manuscript, since no masking was performed

**Additional data files (“source data”)**

- We encourage you to upload relevant additional data files, such as numerical data that are represented as a graph in a figure, or as a summary table
- Where provided, these should be in the most useful format, and they can be uploaded as “Source data” files linked to a main figure or table
- Include model definition files including the full list of parameters used
- Include code used for data analysis (e.g., R, MatLab)
- Avoid stating that data files are “available upon request”

Please indicate the figures or tables for which source data files have been provided:

Data shown in figures is uploaded as a separate excel file for each figure. The excel files contain the information about the sample size, each numerical sample value and group allocation due to treatment.
